# Development and Application of *Pik* Locus-Specific Molecular Markers for Blast Resistance Genes in Yunnan *Japonica* Rice Cultivars

**DOI:** 10.3390/plants14040592

**Published:** 2025-02-15

**Authors:** Pei Liu, Wumin Zhou, Liying Dong, Shufang Liu, Gul Nawaz, Liyu Huang, Qinzhong Yang

**Affiliations:** 1 Key Laboratory of Green Prevention and Control of Agricultural Transboundary Pests of Yunnan Province, Agricultural Environment and Resources Institute, Yunnan Academy of Agricultural Sciences, Kunming 650205, China; liupei@yaas.org.cn (P.L.); zhouwumin97@163.com (W.Z.); dliying72@yaas.org.cn (L.D.); liusf@yaas.org.cn (S.L.); gulnawaz@yaas.org.cn (G.N.); 2 School of Agriculture, Yunnan University, Kunming 650504, China; lyhuang@ynu.edu.cn

**Keywords:** *Oryza sativa* L., *Pik* locus, rice blast, resistance gene, molecular marker

## Abstract

Rice blast, caused by the fungal pathogen *Magnaporthe oryzae*, is one of the most devastating diseases affecting rice production worldwide, resulting in significant yield losses and threatening global food security. The severity of rice blast, particularly in susceptible regions, underscores the urgent need for available effective resistance strategies. In this study, six sets of gene-specific molecular markers for the *Pik* locus associated with rice blast resistance were developed based on publicly available gene sequences. Experimental validation confirmed their high accuracy. During the marker development process, a novel haplotype of the *Pik* locus was identified. This haplotype is characterized by 14 bp mutations and a 9 bp insertion within the coding sequence region when compared to the *Pikh* allele. Subsequently, a molecular marker specific to this haplotype was developed and validated. The application of these seven sets of markers to analyze 163 *japonica* rice cultivars bred in Yunnan Province between 1980 and 2020 revealed that 38.65% of the cultivars carry the *Piks* allele, indicating a low resistance frequency against the rice blast fungus under field conditions. In contrast, only a small proportion of cultivars possess other *Pik* locus alleles, which exhibit higher resistance frequencies. These findings highlight the limited utilization of *Pik* locus genes in *japonica* rice breeding in Yunnan. Furthermore, 21.47% of the cultivars lack any of the aforementioned *Pik* locus alleles, indicating the genetic diversity and complexity of the rice genetic resources of Yunnan Province.

## 1. Introduction

Rice (*Oryza sativa* L.) is a fundamental staple food for approximately half of the global population, rendering it one of the most critical food crops worldwide [1]. However, rice production faces significant challenges, particularly from rice blast disease, caused by the ascomycete fungus *Magnaporthe oryzae* (syn. *Pyricularia oryzae*) [2,3]. This disease is recognized as one of the most destructive fungal diseases in agriculture globally, posing a severe threat to rice cultivation. Statistics indicate that the global economic loss attributed to rice blast exceeds USD 7 billion annually [4]. The rice yield reduction in fields where rice blast occurs is about 10% to 30% on average [5], with severe outbreaks potentially leading to complete crop failure [6]. The most cost-effective, efficient, and environmentally sustainable strategy for mitigating rice blast is to deploy resistance genes to develop resistant rice cultivars [7,8,9].

Through long-term and extensive genetic analysis and research worldwide, nearly 118 rice blast resistance genes have been identified [10], and among them, about 38 genes have been cloned [11,12,13]. Genetic analysis has revealed that most of these resistance genes are dominant, while a few, such as *pi21* and *Pi55*(t), are recessive [14,15]. Structural and functional analysis revealed that with the exception of *Pid2*, *pi21*, and *Ptr*, which encode proteins with unique structures, most resistance genes encode proteins with coiled-coil (CC), nucleotide-binding site (NBS), and leucine-rich repeat (LRR) domains [16,17]. These resistance genes are unevenly distributed across 12 chromosomes of rice, with a concentration on chromosomes 6, 11, and 12, exhibiting a clustered distribution pattern [18].

The accurate identification of resistance genes in rice germplasm is essential for their effective utilization in breeding programs. Traditionally, this has been accomplished by selecting specific pathogenic strains of *M. oryzae* and inoculating rice cultivars to assess resistance or susceptibility, thus inferring the presence of particular resistance genes [19]. However, this kind of work is time-consuming and inefficient, with results often being unreliable due to variability in rice growth conditions and inoculation processes [20]. Compared with inoculation experiments, the application of molecular markers is more convenient and saves time. With the identification and fine mapping of resistance genes, a number of molecular markers closely linked to target resistance genes have been developed and applied in practice, such as molecular marker-assisted selection (MAS) breeding [21]. Despite this, the accuracy of using closely linked markers for identifying target genes could be affected by linkage disequilibrium breakdown, population specificity, genetic recombination, as well as a lack of causal relationship with resistant phenotype. Additionally, closely linked markers often fail to differentiate between alleles at the same locus [8,22]. As rice blast resistance genes continue to be cloned, their complete gene sequences have become available, facilitating the development of gene-specific molecular markers. These markers enable the direct selection of target genes, overcoming the limitations associated with closely linked markers and ensuring greater selection accuracy [23].

The *Pik* locus, located at the terminus of the long arm of chromosome 11, comprises at least seven alleles: *Pik* [24], *Pi1* [25], *Pikm* [26], *Piks* [27,28], *Pikp* [29], *Pikh* [30], and *Pike* [31]. The resistance conferred by genes at the *Pik* locus is governed by two adjacent CC-NBS-LRR genes (such as *Pik-1* and *Pik-2*) with opposite transcriptional orientations [26]. Studies targeting Pikm have found that the nucleic acid polymorphism is primarily associated with *Pikm-1*, with variation concentrated in the CC domain, whereas *Pikm-2* remains more conserved [32]. A mechanistic model of *Pikh-*mediated resistance proposes that *Pikh-1* functions as an adaptor, facilitating the interaction between *AvrPik-h* and *Pikh-2.* Subsequently, *Pikh-2* transduces the signal to trigger *Pikh*-specific resistance [30]. The *Pik* locus is critically important due to the application of its alleles in rice breeding programs aimed at achieving durable resistance against rice blast [33]. The allele *Pik* confers high and stable resistance to a wide range of Chinese rice blast isolates [24]. Likewise, the *Pi1* allele originally derived from the West African cv. LAC23 [25] has exhibited durable and high-level resistance to rice blast [34]. Furthermore, Liu et al. [35] reviewed the broad-spectrum resistance against *M. oryzae* by *Pikh* and *Pi1*.

Yunnan is not only a center of diversity for Asia-cultivated rice (*O. sativa*) but also the origin of rice blast fungus (*M. oryzae*) [36,37], which presents unique opportunities for rice blast resistance breeding. To harness Yunnan’s rich genetic resources, it is imperative to accurately and swiftly identify resistance genes. At the same time, the *Pik* locus offers vital materials for exploitation in resistance breeding. Currently, six alleles at the *Pik* locus, excluding *Pike*, have been cloned and published sequences. However, as new allele sequences are identified, previously developed markers may lose accuracy. In this study, the sequence information of these six alleles was analyzed in order to develop more precise and effective gene-specific molecular markers. These newly developed markers were subsequently applied to assess 163 *japonica* rice cultivars bred in Yunnan from 1980 to 2020, with the goal of determining the distribution of these six resistance genes at the *Pik* locus. The aim of this research is to provide valuable insights and guidance for selecting resistance gene combinations in future rice breeding programs.

## 2. Results

### 2.1. Development and Identification of Resistance Gene-Specific Molecular Markers

#### 2.1.1. Pik

**Figure 1:**
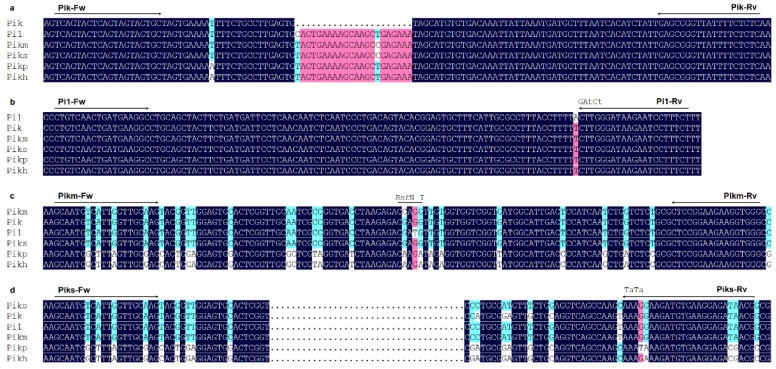
Schematic drawing of primers design for *Pik* locus resistance genes *Pik* (**a**), *Pi1* (**b**), *Pikm* (**c**), and *Piks* (**d**). The solid arrows indicate the location of forward and reverse primers. The uppercase letters represent complementary bases, and the lowercase letters represent introduced mismatched bases in reverse primers.

**Figure 2:**
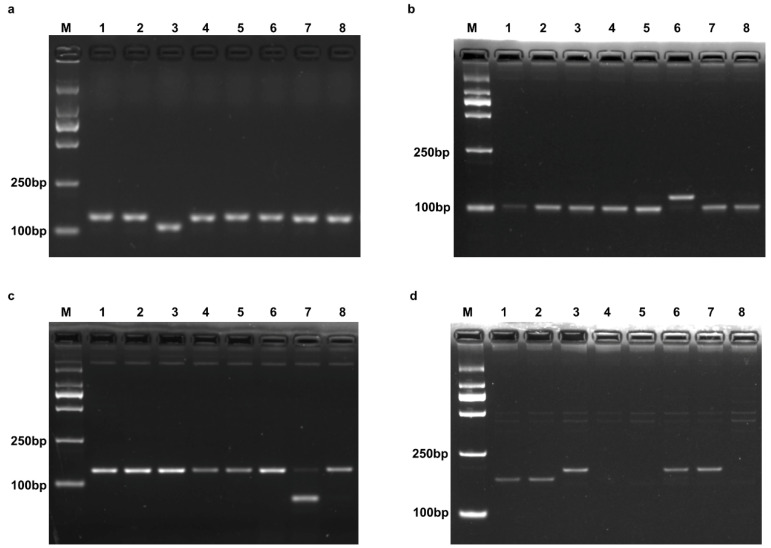
Electrophoresis of amplification product using gene-specific primers of *Pik* (**a**), *Pi1* (**b**), *Pikm* (**c**), and *Piks* (**d**). M, DNA standard molecular weight DL 2000 (Takara). 1–8, monogenic rice lines IRBLks-F5 (*Piks*), IRBLks-S (*Piks*), IRBLk-Ka (*Pik*), IRBLkp-K60 (*Pikp*), IRBLkh-K3 (*Pikh*), IRBL1-CL (*Pi1*), IRBLkm-TS (*Pikm*), and LTH.

Comparison of the gDNA sequence of *Pik* with other alleles revealed a 22-nucleotide deletion located downstream of the coding region of *Pik-1* (Figure 1a). An insertion/deletion (In/Del) marker (Pik-Fw/Rv, Table 1) was developed to detect this deletion and distinguish *Pik* from other alleles. The amplification fragment length of monogenic rice line IRBLk-Ka (*Pik*), using this newly developed marker, is 111 bp, shorter than counterparts of other alleles, including those from Lijiangxintuanheigu (LTH), which were 133 bp (Figure 2a).

#### 2.1.2. Pi1

Sequence alignment of the coding sequence (CDS) of *Pi1-6* with other alleles identified a specific single nucleotide polymorphism (SNP), where *Pi1-6* contains an adenine (A) base, while the others have thymine (T) (Figure 1b). Based on this SNP, a dCAPS marker (Pi1-Fw/Rv, Table 1) was developed, producing an amplification fragment of 120 bp for all monogenic rice lines and LTH. The amplification product was then digested with *Xba* I and analyzed using 2% agarose gel electrophoresis. The amplification product specific to *Pi1* could not be digested with *Xba* I, and its length remained 120 bp. By contrast, the amplification products of other monogenic rice lines and LTH were cleaved with *Xba* I, yielding fragments of 99 bp and 21 bp, which were shorter than the product for *Pi1* (Figure 2b).

#### 2.1.3. Pikm

The sequence alignment of CDS of *Pikm-1-TS* with other alleles identified an SNP where *Pikm* contains a cytosine (C) base, while the other alleles have guanine (G). This C base, along with its flanking sequences of *Pikm-1-TS,* creates a recognition site for the restriction enzyme *Bst*N I (Figure 1c). A CAPS marker (Pikm-Fw/Rv, Table 1) was developed to amplify this fragment, which is 134 bp in length. Following amplification and digestion with *Bst*N I, the amplification product of *Pikm* cleaved into two fragments of equal length (67 bp), while the amplification products of other monogenic rice lines and LTH remained uncut at 134 bp (Figure 2c).

#### 2.1.4. Piks

The polymorphism in the CDS between *Piks-1* and its counterparts (*Pikp*, *Pikh,* and the allele in LTH) led to the development of a forward primer by introducing 4, 4, and 6 mismatched bases compared to the target sequence of *Pikp*, *Pikh*, and LTH (Figure 1d). Additionally, an SNP site, which is C for *Piks* and T for *Pik*, *Pi1*, and *Pikm*, located in the reverse primer region, enabled the development of a dCAPS marker (Piks-Fw/Rv, Figure 1d). Due to the introduction of mismatched bases, it was not possible for the dCAPS marker for *Piks* to amplify products representing *Pikp*, *Pikh*, and LTH. The remaining amplification product specific to *Piks* is 189 bp in length. Following digestion with *Nde* I, only the amplification product representing *Piks* is cleaved, resulting in a fragment of 165 bp, which is significantly shorter than those of other alleles when analyzed by 2% agarose gel electrophoresis (Figure 2d).

#### 2.1.5. Pikp and Pikh

**Figure 3:**
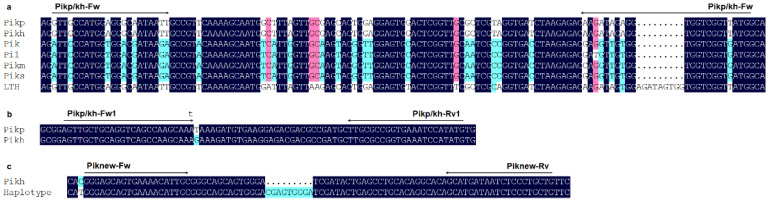
Schematic drawing of primers design for *Pik* locus resistance genes *Pikp* and *Pikh*. The sequence differences are shown in (**a**). The lowercase letter represents the mismatched base (A to T) in the forward primer (**b**). The 9 bp insertion located in the CDS of the novel *Pik* locus haplotype, compared with *Pikh*, is illustrated in (**c**).

**Figure 4:**
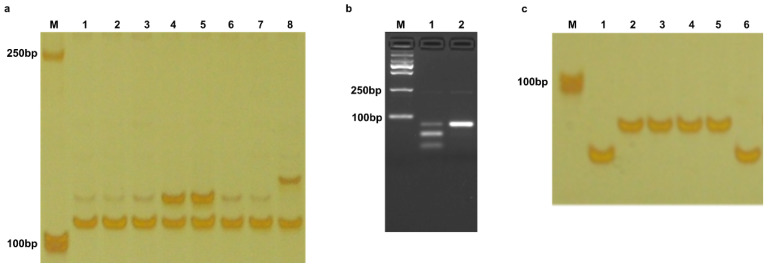
The electrophoresis of amplification product using primers Pikp/kh-Fw/Rv and TBC-Fw/RV; (**a**), amplification product using Pikp/kh-Fw1/Rv1 after *Mse* I digestion (**b**), and amplification product using Piknew-Fw/Rv (**c**). M, DNA standard molecular weight DL 2000 (Takara). 1–8, monogenic rice lines IRBLks-F5 (*Piks*), IRBLks-S (*Piks*), IRBLk-Ka (*Pik*), IRBLkp-K60 (*Pikp*), IRBLkh-K3 (*Pikh*), IRBL1-CL (*Pi1*), IRBLkm-TS (*Pikm*), and LTH in panel a. 1–2, monogenic rice lines IRBLkp-K60 (*Pikp*) and IRBLkh-K3 (*Pikh*) in panel b. 1–6, monogenic rice line IRBLkh-K3 (*Pikh*), Chugeng 5, Yungeng 37, Hexi 3, Ligeng 15, and LTH.

Due to the minimal differences in coding sequence between *Pikp* and *Pikh* and interference from the allele present in LTH, we initially designed a co-dominant marker; this marker introduces 7 and 6 mismatched bases, compared to *Pik*, *Pi1*, *Pikm*, and *Piks*, in the forward and reverse primers (Pikp/kh-Fw/Rv, Table 1), respectively, to distinguish *Pikp*/*Pikh* from *Pik*, *Pi1*, *Pikm*, *Piks*, and LTH (Figure 3a). With this marker, a 122 bp amplification product will be obtained, which represents *Pikp* or *Pikh*. Due to the introduced mismatched bases, no amplification could be observed with other monogenic rice lines as samples. A 9 bp longer fragment was found in the amplification region, so the product representing LTH is 131 bp (Figure 4a). To prevent false-negative results, a housekeeping gene, *TBC*, with its specific marker (TBC-Fw/Rv, Table 1), was incorporated to confirm the PCR reaction.

An SNP site between *Pikp* and *Pikh* was used to develop a dCAPS marker (Pikp/kh-Fw1/Rv1, Table 1), which introduces restriction endonuclease *Mse* I site in the amplification product of *Pikp* (Figure 3b). Upon digestion, the *Pikp* product is cleaved into 51 bp and 25 bp fragments, while the *Pikh* product remains 79 bp (Figure 4b).

#### 2.1.6. A Novel Haplotype of Pik Locus

Using the newly developed dCAPS marker Pikp/kh-Fw1/Rv1 to analyze the 163 *japonica* rice cultivars, we unexpectedly identified a novel haplotype at the *Pik* locus. The amplification product of this haplotype is approximately 79 bp long and cannot be digested by *Mse* I, displaying the same digestion pattern as *Pikh*. However, Sanger sequencing demonstrated that there are 14 bp mutations and a 9 bp insertion distributing in the CDS region of this haplotype compared with *Pikh* (Figure 3c). To further differentiate the novel haplotype from *Pikh*, we conducted additional amplification using the newly developed marker Piknew-Fw/Rv (Table 1), followed by 8% acrylamide gel electrophoresis analysis. The results showed a significant difference between this novel haplotype and *Pikh,* enabling a clear distinction (Figure 4c).

### 2.2. Determination of 163 Japonica Rice Cultivars with Pik Allele-Specific Molecular Markers

The newly developed molecular markers were employed to analyze 163 *japonica* rice cultivars bred in Yunnan from 1980 to 2020, to determine the distribution of the *Pik* alleles in these cultivars. The findings are summarized in Table 2.

**Figure 5:**
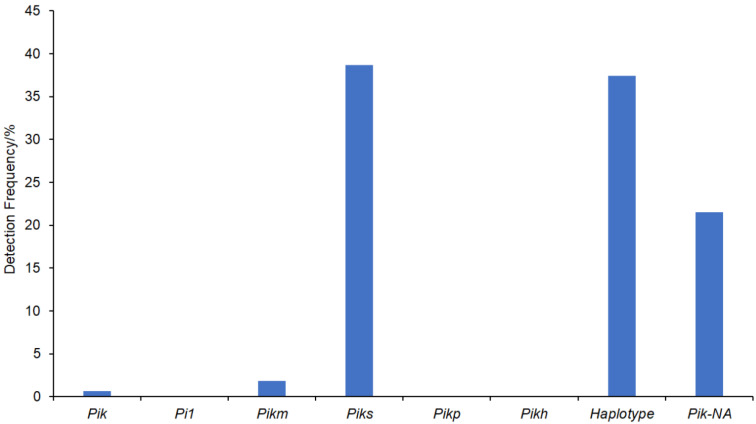
The detection frequency of *Pik* locus genes in 163 *japonica* rice cultivars.

Among six alleles at the *Pik* locus, *Piks* is the most widely distributed, with 63 cultivars containing *Piks*, which corresponds to a detection frequency of 38.65%. In contrast, *Pikm* and *Pik* were detected in only three and one cultivars, with corresponding detection frequencies of 1.84% and 0.61%, respectively. No cultivars were found to carry *Pi1*, *Pikp*, and *Pikh.* Interestingly, 61 cultivars were identified as carrying the novel *Pik* locus haplotype through detection with Pikp/kh-Fw1/Rv1, representing a detection frequency of 37.42%. The remaining 35 cultivars did not contain any of these 7 *Pik* locus alleles/haplotypes, with a detection frequency is 21.47%. This reveals an uncovered genetic diversity of these cultivars (Figure 5).

These 163 cultivars can be divided into seven major breeding series based on their origins of development: ‘Chugeng’, ‘Yungeng’, ‘Xiugeng’, ‘Ligeng’, ‘Fengdao’, ‘Jinggeng’, and ‘Hexi’. These series were developed by scientific institutions across different regions of Yunnan, reflecting the diversity of rice genetic resources in the region. The results indicated that *Piks* is distributed across all seven series. *Pikm* was detected in cultivars of the ‘Chugeng’ and ‘Xiugeng’ series, while *Pik* was identified in cultivars of ‘Jinggeng’. The novel *Pik* locus haplotype was found in all five series except for ‘Jinggeng’ and ‘Xiugeng’, with the most widespread distribution being in ‘Chugeng’, ‘Yungeng’, and ‘Hexi’. Interestingly, cultivars lacking any of these seven *Pik* alleles/haplotypes were found in all series, underscoring the widespread occurrence of this genetic trait (Table 2).

## 3. Discussion

In this study, we successfully developed gene-specific molecular markers for six alleles at the *Pik* locus using publicly accessible gene sequences. These markers have been proven effective in accurately identifying target genes within rice genetic resources. Furthermore, a novel haplotype of *Pik* locus was found during the process of marker development. This new haplotype contains nucleotide mutations in its CDS region compared to known functional alleles, resulting in changes to the corresponding amino acid sequence, including a 9 bp insertion that encodes three additional amino acids. Based on these unique characteristics, we have developed haplotype-specific molecular markers to analyze the distribution of this new haplotype among improved rice cultivars of Yunnan.

The application of seven sets of specific markers developed by this study to detect 163 developed *japonica* rice cultivars from the 1980s to 2020s in Yunnan revealed a limited utilization of *Pik* locus resistance genes. Specifically, 38.65% of the cultivars, spanning all seven breeding series, carry the *Piks* allele, which confers a low resistance frequency against *M. oryzae* in the field. In contrast, the remaining five alleles of the *Pik* locus are rarely utilized, with detection frequencies of 1.84% for *Pikm*, 0.61% for *Pik*, and 0% for *Pi1*, *Pikp*, and *Pikh*, respectively. This underutilization of *Pik* locus resistance genes restricts their potential application in rice breeding programs. Moreover, the long-term reliance on a single resistance gene can significantly increase selection pressure and accelerate the breakdown of resistance [38]. Interestingly, 21.47% of the cultivars, distributed across all seven breeding series, do not carry any of the known *Pik* locus alleles. This observation highlights the genetic diversity of Yunnan’s rice genetic resources and suggests the presence of unknown genes/alleles that may harbor new resistance functions. Despite the high sequence similarity among the coding sequences of *Pik* locus alleles, their resistance functions differ significantly. Chen et al. [31] tested the resistance spectra of these six alleles of *Pik* locus with 215 *M. oryzae* isolates collected from different regions of China. The results showed a low resistance spectrum of *Piks* (with a resistance frequency of 13.0%), medium resistance spectrum of *Pikp* (31.6%), *Pik* (39.1%), *Pi1* (48.4%), *Pikm* (50.2%), and *Pikh* (51.6%). However, 38.65% of the 163 *japonica* rice cultivars bred in Yunnan carry *Piks*, while the other alleles were rarely utilized. This imbalance further underscores the limitations of current *Pik* locus allele usage in rice breeding. The development of these sets of gene-specific markers will facilitate the rapid identification of *Pik* locus alleles carried by rice genetic resources, expand the utilization of *Pik* locus alleles, and facilitate the pyramiding of *Pik* genes with other resistance genes to enhance both the resistance spectrum and the longevity of resistance in subsequent rice breeding efforts.

By integrating the latest sequence information, we have developed six sets of accurate and practical gene-specific markers for detecting alleles at the *Pik* locus in Yunnan rice cultivars. Our findings revealed that alleles at the *Pik* locus are limited in improved cultivars. This indicates that alleles such as *Pi1*, *Pikm*, and *Pikh,* which exhibit good resistance against *M. oryzae* [39], could be strategically utilized in disease-resistant rice breeding in Yunnan to broaden the resistant spectrum. Notably, we identified a novel haplotype at the *Pik* locus, with 37.42% of the improved Yunnan cultivars carrying this new haplotype. Further investigations into whether this haplotype represents a functional resistance allele are currently ongoing.

## 4. Materials and Methods

### 4.1. Rice Cultivars

A total of 163 *japonica* rice cultivars bred in Yunnan from 1980 to 2020 (Table 2), monogenic rice lines IRBLk-Ka (*Pik*), IRBL1-CL (*Pi1*), IRBLkm-Ts (*Pikm*), IRBLks-F5 (*Piks*), IRBLks-S (*Piks*), IRBLkp-K60 (*Pikp*), IRBLkh-K3 (*Pikh*), and LTH, were collected and preserved by the laboratory.

### 4.2. Development of Gene-Specific Molecular Markers

The sequences of cloned resistance genes *Pik* (NCBI accession number AB616659), *Pi1* (HQ606329), *Pikm* (AB462256), *Piks* (HQ662329), *Pikp* (HM035360), and *Pikh* (HQ662330), as well as housekeeping gene *TBC* (LOC_Os09g34040, [40]) were utilized for the development of relevant molecular markers. Primers and CAPS markers were designed by using SnapGene V4.1.8. dCAPS markers were generated through dCAPS Finder 2.0 (http://helix.wustl.edu/dcaps/ (accessed on 12 January 2025), [41]). The primers and markers used are listed in Table 1.

### 4.3. Genomic DNA Extraction

Genomic DNA (gDNA) was extracted from seedling leaves using the CTAB method described by Warude et al. [42]. The integrity of the extracted gDNA was assessed via 1% agarose gel electrophoresis. The gDNA concentration of each sample was measured by Nanodrop^TM^ 1000 spectrophotometer (Thermo Scientific, Waltham, MA, USA) and then adjusted to a working concentration of 20 ng/μL with 0.1× TE buffer. The samples were then stored at −20 °C.

### 4.4. PCR Amplification and Electrophoresis

Each 20.0 μL PCR reaction mixture was prepared with the following components: 10.0 μL 2× Es Taq MasterMix (Dye) (CWBIO, Taizhou, China), 1.0 μL gDNA (20 ng/μL), 1.0 μL each of forward and reserve primers, and 7.0 μL of ddH_2_O. The PCR protocol consisted of an initial denaturation at 95 °C for 3 min, followed by 34 cycles of denaturation at 95 °C for 30 s, annealing at temperatures specified in Table 1 for 30 s, and extension at 72 °C for 30 s. The PCR products targeting the *Pik* gene were analyzed using 2% agarose gel electrophoresis; PCR products amplified for *Pi1*, *Pikm*, and *Piks* were subsequently digested with relevant restriction enzymes and analyzed with 2% agarose gel electrophoresis. PCR products generated using Pikp/kh-Fw and Pikp/kh-Rv, targeting *Pikp*/*Pikh*, were analyzed by 8% acrylamide gel electrophoresis to distinguish *Pikp* and *Pikh* with *Pik*, *Pi1*, *Pikm*, and *Piks*, with *TBC* used as an internal control to confirm successful amplifications. To further distinguish *Pikp* from *Pikh*, PCR products amplified with Pikp/kh-Fw1 and Pikp/kh-Rv1 were digested with *Mse* I and analyzed with 2% agarose gel electrophoresis. Additionally, PCR products amplified using Piknew-Fw and Piknew-Rv primers were analyzed by 8% acrylamide gel electrophoresis to identify a novel *Pik* locus haplotype distinct from *Pikh*.

## Figures and Tables

**Table 1:** List of primers used in this study.

Target Gene	Marker Name	Sequence (5′-3′)	Fragment Length/bp	Enzyme Site	Annealing Temperature/°C	Marker Type
*Pik*	Pik-Fw	TCAGTACTCAGTAGTAGTGC	111 ^a^/133 ^b^	-	55	InDel
	Pik-Rv	TGAGAGAAAATAACCCGCTC				
*Pi1*	Pi1-Fw	CTGTCAACTGATGAAGGC	120/99 + 21	*Xba* I	55	dCAPS
	Pi1-Rv	AGAAAGGATTCTTATCtCtAG				
*Pikm*	Pikm-Fw	GCAATGTCATTGGTTGCAAG	67 + 67/134	*Bst*N I	58	CAPS
	Pikm-Rv	CCCACCTTCTTCCGGAG				
*Piks*	Piks-Fw	GCAATGTCATTGGTTGCAAG	167 + 22/189	*Nde* I	53	dCAPS
	Piks-Rv	CGTTATCTCCTTCACATCTTCaTaT				
*Pikp*/*Pikh*	Pikp/kh-Fw	GTTGCCATGGAGGGCAATAATT	122/-	-	60	dominant
	Pikp/kh-Rv	CCATAACCGACCACCTCTATCTT				
	TBC-Fw	TGGTCATGTTCCTTCAGCAC	111 ^c^	-	60	internal control
	TBC-Rv	GACTTGGCGAGCTTTTGAAC				
	Pikp/kh-Fw1	AGTTGCTGCAGGTCAGCCAAGCAAt	51 + 25/76	*Mse* I	60	dCAPS
	Pikp/kh-Rv1	CATATGGATTTCACCGGCGCAA				
	Piknew-Fw	GGGAGCAGTGAAAACATTGC	89/80	-	55	InDel
	Piknew-Rv	ACAGCAGGGAGATTATCATGC				

^a^: the fragment length of the target resistance gene after amplification and/or digestion. ^b^: the fragment length of the allele gene after amplification and/or digestion. ^c^: the fragment length of internal control gene *TBC* after amplification for all resistance genes. Lowercase letters indicate introduced mismatch nucleotide bases.

**Table 2:** 163 *japonica* rice cultivars bred in Yunnan Province from 1980 to 2020 containing *Pik* locus resistance genes.

No.	Name	Year of Registration	*Pik*	*Pi1*	*Pikm*	*Piks*	*Pikp*	*Pikh*	Novel Haplotype
1	Chugeng 3	1983	-	-	-	+	-	-	-
2	Chugeng 4	1985	-	-	+	-	-	-	-
3	Chugeng 5	1986	-	-	-	-	-	-	+
4	Chugeng 6	1990	-	-	-	-	-	-	+
5	Chugeng 7	1991	-	-	-	-	-	-	+
6	Chugeng 8	1990	-	-	-	-	-	-	+
7	Chugeng 9	-	-	-	-	-	-	-	+
8	Chugeng 13	-	-	-	-	-	-	-	+
9	Chugeng 14	1995	-	-	-	-	-	-	+
10	Chugeng 15	-	-	-	-	-	-	-	+
11	Chugeng 17	1997	-	-	-	+	-	-	-
12	Chugeng 18	-	-	-	+	-	-	-	-
13	Chugeng 19	-	-	-	-	+	-	-	-
14	Chugeng 21	-	-	-	-	-	-	-	+
15	Chugeng 22	1999	-	-	-	-	-	-	+
16	Chugeng 23	1999	-	-	-	+	-	-	-
17	Chugeng 25	2002	-	-	-	-	-	-	+
18	Chugeng 26	2005	-	-	-	-	-	-	+
19	Chugeng 27	2005	-	-	-	-	-	-	+
20	Chugeng 28	2007	-	-	-	+	-	-	-
21	Chugeng 29	2007	-	-	-	+	-	-	-
22	Chugeng 30	2007	-	-	-	+	-	-	-
23	Chugeng 31	2010	-	-	-	+	-	-	-
24	Chugeng 32	2011	-	-	-	+	-	-	-
25	Chugeng 34	-	-	-	-	+	-	-	-
26	Chugeng 35	-	-	-	-	-	-	-	+
27	Chugeng 36	-	-	-	-	+	-	-	-
28	Chugeng 37	2014	-	-	-	-	-	-	+
29	Chugeng 38	2014	-	-	-	-	-	-	+
30	Chugeng 40	2015	-	-	-	-	-	-	+
31	Chugeng 41	2016	-	-	-	-	-	-	+
32	Chugeng 42	2016	-	-	-	+	-	-	-
33	Chugeng 45	2017	-	-	-	-	-	-	+
34	Chugeng 48	2019	-	-	-	-	-	-	-
35	Chugeng 53	-	-	-	-	-	-	-	+
36	Chugeng 54	-	-	-	-	+	-	-	-
37	Hongza 135	1989	-	-	-	-	-	-	-
38	Yundao 1	2005	-	-	-	+	-	-	-
39	DJY 5	2005	-	-	-	-	-	-	-
40	Dian 4	2001	-	-	-	-	-	-	+
41	Yinguang	2001	-	-	-	+	-	-	-
42	Yunzigeng 41	2012	-	-	-	+	-	-	-
43	Yungeng 2	-	-	-	-	+	-	-	-
44	Yungeng 3	-	-	-	-	+	-	-	-
45	Yungeng 4	2001	-	-	-	-	-	-	+
46	Yungeng 5	-	-	-	-	+	-	-	-
47	Yungeng 6	-	-	-	-	+	-	-	-
48	Yungeng 7	-	-	-	-	+	-	-	-
49	Yungeng 10	-	-	-	-	+	-	-	-
50	Yungeng 12	2005	-	-	-	-	-	-	+
51	Yungeng 14	-	-	-	-	-	-	-	+
52	Yungeng 16	-	-	-	-	-	-	-	-
53	Yungeng 17	-	-	-	-	-	-	-	+
54	Yungeng 18	-	-	-	-	-	-	-	+
55	Yungeng 19	2010	-	-	-	-	-	-	+
56	Yungeng 20	2011	-	-	-	-	-	-	+
57	Yungeng 21	-	-	-	-	-	-	-	+
58	Yungeng 24	2007	-	-	-	+	-	-	-
59	Yungeng 25	2007	-	-	-	-	-	-	+
60	Yungeng 26	2010	-	-	-	-	-	-	+
61	Yungeng 29	2011	-	-	-	-	-	-	+
62	Yungeng 30	2011	-	-	-	-	-	-	+
63	Yungeng 31	2011	-	-	-	-	-	-	+
64	Yungeng 32	2011	-	-	-	-	-	-	+
65	Yungeng 35	2014	-	-	-	+	-	-	-
66	Yungeng 37	-	-	-	-	-	-	-	+
67	Yungeng 38	2014	-	-	-	-	-	-	+
68	Yungeng 39	2014	-	-	-	+	-	-	-
69	Yungeng 42	2016	-	-	-	-	-	-	+
70	Yungeng 43	2016	-	-	-	-	-	-	+
71	Yungeng 46	2018	-	-	-	-	-	-	+
72	Yungeng 48	-	-	-	-	-	-	-	+
73	Yungeng 135	-	-	-	-	+	-	-	-
74	Yungeng 136	1983	-	-	-	-	-	-	-
75	Yungengyou 1	2004	-	-	-	+	-	-	-
76	Yungengyou 5	2005	-	-	-	+	-	-	-
77	Hexi 1	-	-	-	-	+	-	-	-
78	Hexi 2	1991	-	-	-	+	-	-	-
79	Hexi 3	-	-	-	-	-	-	-	+
80	Hexi 4	1990	-	-	-	-	-	-	+
81	Hexi 5	1990	-	-	-	+	-	-	-
82	Hexi 6	-	-	-	-	+	-	-	-
83	Hexi 8	-	-	-	-	-	-	-	+
84	Hexi 9	-	-	-	-	+	-	-	-
85	Hexi 10	1990	-	-	-	-	-	-	+
86	Hexi 12	-	-	-	-	-	-	-	+
87	Hexi 13	-	-	-	-	+	-	-	-
88	Hexi 14	-	-	-	-	+	-	-	-
89	Hexi 15	1993	-	-	-	-	-	-	-
90	Hexi 16	-	-	-	-	+	-	-	-
91	Hexi 17	-	-	-	-	-	-	-	+
92	Hexi 20	-	-	-	-	-	-	-	+
93	Hexi 22	1991	-	-	-	-	-	-	+
94	Hexi 23	-	-	-	-	+	-	-	-
95	Hexi 24	1993	-	-	-	-	-	-	+
96	Hexi 25	1993	-	-	-	-	-	-	-
97	Hexi 28	-	-	-	-	-	-	-	-
98	Hexi 30	1993	-	-	-	-	-	-	+
99	Hexi 32	-	-	-	-	-	-	-	+
100	Hexi 34	1997	-	-	-	+	-	-	-
101	Hexi 35	1997	-	-	-	-	-	-	-
102	Hexi 38	-	-	-	-	+	-	-	-
103	Hexi 40	1999	-	-	-	+	-	-	-
104	Hexi 41	1999	-	-	-	-	-	-	+
105	Hexi 42	1999	-	-	-	-	-	-	+
106	Fengdao 9	1997	-	-	-	-	-	-	-
107	Fengdao 11	1999	-	-	-	+	-	-	-
108	Fengdao 12	-	-	-	-	+	-	-	-
109	Fengdao 14	2001	-	-	-	-	-	-	-
110	Fengdao 15	2002	-	-	-	-	-	-	-
111	Fengdao 16	2004	-	-	-	-	-	-	-
112	Fengdao 17	2003	-	-	-	+	-	-	-
113	Fengdao 18	2005	-	-	-	-	-	-	-
114	Fengdao 19	2006	-	-	-	-	-	-	-
115	Fengdao 20	2006	-	-	-	-	-	-	-
116	Fengdao 21	2007	-	-	-	+	-	-	-
117	Fengdao 23	2010	-	-	-	-	-	-	+
118	Fengdao 29	2014	-	-	-	-	-	-	-
119	Fengdao 30	2017	-	-	-	+	-	-	-
120	Jinggeng 3	2005	-	-	-	-	-	-	-
121	Jinggeng 6	2009	-	-	-	-	-	-	-
122	Dianyuyi	1983	-	-	-	+	-	-	-
123	Jinggeng 8	2001	-	-	-	+	-	-	-
124	Jinggeng 11	2007	-	-	-	+	-	-	-
125	Jinggeng 12	2007	-	-	-	+	-	-	-
126	Jinggeng 13	2007	-	-	-	+	-	-	-
127	Jinggeng 14	2007	-	-	-	+	-	-	-
128	Jinggeng 16	2010	-	-	-	+	-	-	-
129	Jinggeng 17	2010	-	-	-	+	-	-	-
130	Jinggeng 18	2010	-	-	-	+	-	-	-
131	Jinggeng 26	2014	+	-	-	-	-	-	-
132	Jingdao 1	2018	-	-	-	-	-	-	-
133	Jingdao 5	2018	-	-	-	-	-	-	+
134	Jinggengyou 1	2003	-	-	-	-	-	-	-
135	Jinggengyou 2	2005	-	-	-	-	-	-	-
136	Jinggengyou 3	2005	-	-	-	-	-	-	+
137	Ligeng 4	-	-	-	-	-	-	-	-
138	Ligeng 6	2004	-	-	-	-	-	-	-
139	Ligeng 9	2012	-	-	-	-	-	-	-
140	Ligeng 10	2009	-	-	-	-	-	-	-
141	Ligeng 11	2010	-	-	-	-	-	-	-
142	Ligeng 15	2014	-	-	-	-	-	-	+
143	Ligeng 18	2018	-	-	-	+	-	-	-
144	Ligeng 22	2019	-	-	-	+	-	-	-
145	Ligeng 23	2019	-	-	-	-	-	-	-
146	Ligeng 314	2007	-	-	-	-	-	-	-
147	Xiugeng 12	2011	-	-	-	-	-	-	-
148	Xiugeng 18	2015	-	-	+	-	-	-	-
149	Xiugeng 22	2013	-	-	-	-	-	-	-
150	Xiugeng 26	2018	-	-	-	-	-	-	-
151	Xiugeng 28	2018	-	-	-	+	-	-	-
152	Xiugeng 29	2020	-	-	-	+	-	-	-
153	Xiu 87-15	2003	-	-	-	+	-	-	-
154	Xiu 191-7	2011	-	-	-	-	-	-	-
155	Changgeng 8	2004	-	-	-	+	-	-	-
156	Changgeng 9	2007	-	-	-	-	-	-	-
157	Longke 16	2015	-	-	-	-	-	-	+
158	Yugeng 24	2018	-	-	-	+	-	-	-
159	Yugeng 25	2019	-	-	-	-	-	-	+
160	Tageng 3	2014	-	-	-	+	-	-	-
161	Niangeng 7	1993	-	-	-	+	-	-	-
162	Jinrui 4	2019	-	-	-	-	-	-	+
163	Jinning 78-102	-	-	-	-	-	-	-	-

“-“: refers to cultivars that lack the resistance gene; “+”: refers to cultivars that contain the resistance gene.

## Data Availability

The data that support the findings of this study are accessible from the corresponding author upon reasonable request.
